# Future directions in regulatory affairs

**DOI:** 10.3389/fmed.2022.1082384

**Published:** 2023-01-09

**Authors:** Orin Chisholm, Helen Critchley

**Affiliations:** ^1^Faculty of Medicine and Health, Sydney Pharmacy School, The University of Sydney, Sydney, NSW, Australia; ^2^Sanofi, Macquarie Park, NSW, Australia

**Keywords:** regulatory affairs, regulatory science, drug development, future trends, digital disruption, skills

## Abstract

The field of regulatory affairs deals with the regulatory requirements for marketing authorization of therapeutic products. This field is facing a myriad of forces impacting all aspects of the development, regulation and value proposition of new therapeutic products. Changes in global megatrends, such as geopolitical shifts and the rise of the green economy, have emphasized the importance of manufacturing and supply chain security, and reducing the environmental impacts of product development. Rapid changes due to advances in science, digital disruption, a renewed focus on the centrality of the patient in all stages of therapeutic product development and greater collaboration between national regulatory authorities have been accelerated by the COVID-19 pandemic. This article will discuss the various trends that are impacting the development of new therapies for alleviating disease and how these trends therefore impact on the role of the regulatory affairs professional. We discuss some of the challenges and provide insights for the regulatory professional to remain at the forefront of these trends and prepare for their impacts on their work.

## 1. Introduction

Digital disruption is affecting all aspects of drug development, including the way medicinal products are regulated. At the same time, advances in science have fueled a large increase in the number of cell and gene therapies coming to market and are delivering more benefits for patients (1, 2). The rise in patient input into all aspects of drug development, including regulatory review, has also impacted medicinal product regulation (3). The increasing integration of real-world evidence will enable medicines to reach the market at an earlier stage of development due to faster clinical trials and lead regulators to place greater emphasis on post-market regulation. The clinical trial enterprise is integrating more modeling, newer statistical methodology, and artificial intelligence to improve efficiencies in this stage of development (4, 5). Manufacturing is utilizing digital twins to test out new systems; it is becoming more integrated, with internet-of-things, robotics and continuous manufacturing processes becoming more routine (6). As well, regulatory agencies are collaborating more and developing work-sharing, reliance and collaborative reviews to facilitate the review of these innovative products coming through the regulatory system (7, 8). All these changes require a workforce that is agile, digitally savvy and able to learn and adapt their work processes to meet these new trends. This article will examine the various trends that are impacting the development of new therapies for alleviating disease and how these trends therefore impact on the role of the regulatory affairs professional. Our aim is not to provide a full critique as to the benefits and risks of these developments but rather to alert the regulatory professional to these trends and the need to monitor these developments. We provide insights that regulatory professionals may consider in their professional development programs to ensure they are able to adapt to these new trends and successfully navigate their future careers.

## 2. Megatrends

**FIGURE 1:**
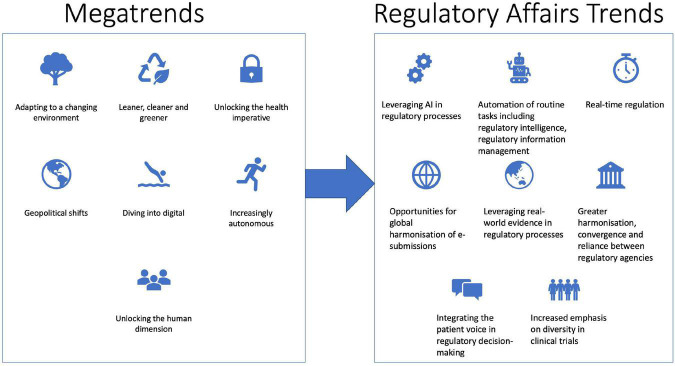
Trends impacting the future of regulatory affairs.

Understanding changes in global megatrends can help regulatory affairs professionals navigate the future impacts on their roles. Megatrends are global trends that may unfold over several years and have the potential to have substantial transformative impacts on society (9, 10). The Australian Commonwealth Scientific and Industrial Research Organisation (CSIRO) has recently updated their global megatrend list to include the following: adapting to a changing environment; leaner cleaner and greener; unlocking the health imperative; geopolitical shifts; diving into digital; increasingly autonomous and unlocking the human dimension (9) (Figure 1). Such megatrends may impact the future of medicine, therapeutic product development and the way that regulatory professionals perform their work. Climate change concerns are impacting manufacturing with an emphasis on sustainable processes, reduced environmental impact and a move toward a circular manufacturing economy (11). Geopolitical uncertainty is being reflected by the increasing drive of governments to manufacture critical pharmaceutical products locally, after the COVID-19 pandemic highlighted risks to supply chains (12). The COVID-19 pandemic has highlighted the risks of infectious diseases, which are likely to increase with climate change. Antimicrobial resistance, the growing chronic health burden of an aging population, increased stressors on mental health and budget constraints on healthcare spending will all impact the pharmaceutical industry. More positively, the promise of precision medicine, increased digital integration across the healthcare system, a move toward a learning healthcare system and an emphasis on wellbeing and preventative medicine will lead to future improvements in healthcare and opportunities for industry (13–15). Arguably, digital disruption, the rise in AI and the human dimension will have the greatest impact on the way regulatory affairs professionals work. Some of the future trends in the regulatory affairs profession that we have identified include leveraging big data, Artificial Intelligence (AI) and machine learning (ML) in regulatory processes, which will facilitate real-time regulation, the utilization of real-world evidence and the increasing role of patient preferences in regulatory decision-making, and an increase in global harmonization, convergence and reliance between national regulatory authorities (16) (Figure 1).

## 3. Digital disruption

Digital disruption is beginning to impact all aspects of the drug development process from early-stage discovery and validation of target molecules, then optimization of the candidate drug structure through the manufacturing process to the regulatory approvals by national regulatory authorities. One of the great challenges in early-stage drug discovery is determining the structure of target proteins and then identifying and optimizing appropriate drug candidates that can interact with these proteins to block or alter their function.

AI and ML are playing an increasingly important role in drug discovery (17). Their application will reduce the failure rate in early-stage drug discovery and development and speed up this step in the pathway (18–20). It should also reduce the risks and cost of drug development. In 2018 and again in 2020, Alphabet’s DeepMind AI predicted with overwhelming success the 3-dimensional (3D) structure of several proteins in the biennial Critical Assessment of Structure Prediction (CASP) competition (21). Since then, DeepMind has released an open source AlphaFold protein structure database which researchers and drug developers can use to determine the 3D structure of potential targets for drug development (18). The utilization of AI/ML systems, such as PandaOmics and Chemistry42, have been used more recently to not only discover novel targets but also accelerate the identification of lead candidate molecules (19, 20).

Digital twins are virtual representations of the physical asset or process that can replicate the behavior of that actual asset or process (22). Digital twins can speed up pharmaceutical manufacturing by simulating process flows before they are implemented to ensure optimisation of the process and by facilitating technology transfer by testing the new manufacturing plant before it is built and having staff train on the digital twin environment before entering the actual manufacturing plant (22, 23). Digital twin models have already been used by pharmaceutical companies, for example, GSK used digital twins to optimize their vaccine development and production processes (24).

As industry increasingly moves toward the new Pharma 4.0, the use of digital twins will increase, as part of the suite of innovations streamlining the manufacturing process by greater integration of digitization and automation (6, 25). Industry 4.0, or the fourth industrial revolution, is the integration of physical and cyber systems (26) while Pharma 4.0 encompasses digitally integrated manufacturing but ensures data integrity by design and includes organization, culture and processes, ensuring compliance with current GMP standards and harmonized guidelines such as those developed by the International Council for Harmonisation of Technical Requirements for Pharmaceuticals for Human Use (ICH) (26, 27). It will allow real-time data submission to regulatory agencies via cloud-based systems such as Accumulus Synergy (28). The Accumulus Synergy platform is designed to hold company-specific spaces where companies can work on their data packages, health authority specific spaces where NRAs can work together on their reviews of product data and share their analyses of these data, and spaces where sponsors and regulators can communicate, all protected by data privacy and cyber-security (28, 29).

Another use of digital twins is modeling disease progression, and this will lead to digital twin control groups in clinical trials, reducing ethical and operational concerns with control cohorts (30–32). As the number of digital twins of individuals increase, there will be a greater ability to predict medicine effects before administering any medication to the patient. Thus, quality use of medicines will be enhanced by enabling testing of the optimal drug, dose, timing and route of administration in a person’s digital twin first, reducing the actual harm that a patient may experience. The use of digital twins will facilitate the growth of precision medicine (32, 33) and lead to greater use of “virtual” or “*in silico*” clinical trials, streamlining the clinical trials enterprise (34).

The use of “virtual” or “*in silico*” clinical trials will impact the regulatory professional since the integrity of the data will become paramount. Digitization of all aspects of regulatory operations will occur through initiatives such as the implementation of the International Organization for Standardization’s (ISO) Identification of Medicinal Products (IDMP) standards which require substance, product, organization and referential (SPOR) data management (35). This relies on an increasing trend toward structured data formats, which will enable real-time exchange of data with national regulatory authorities via cloud-based platforms such as Accumulus Synergy. So, in the future, regulatory professionals will contribute increasingly to data flows rather than document flows, requiring regulatory professionals to upskill in digital literacy (29, 36).

The flow of manufacturing information, such as in-process release testing, specification testing and batch release data in a structured format collected and stored in data lakes will facilitate the collection and exchange of data for regulatory compliance requirements (37). This allows for more efficient transfer of data into the current eCTD format for regulatory submissions by automating eCTD compilation. Currently eCTD writing still requires human oversight and interpretation of the data presented in the documents, which are in portable document format (pdf) that does not enable automated traceability back to original data sources or mining of the data to gain greater insights (38). Structured content and data management systems have the potential to further streamline data handling and the authoring and publication of regulatory documents. To date, companies have been developing bespoke in-house structured content management systems, particularly for clinical data, but common methodologies will be required across companies and regulatory authorities to enable rapid exchange of data and documents (29, 37, 38). Continued evolution of initiatives such as Transcelerate’s Common Protocol Template, and the FDA’s Knowledge-Aided Assessment and Structured Application (KASA), standardizing product quality/chemistry-manufacturing-controls (CMC) data and widespread adoption of the ICH Q12: *Technical and Regulatory Considerations for Pharmaceutical Product Lifecycle Management* guideline will facilitate the move toward use of more structured formats for both clinical and CMC data packages (37, 38). The implementation of structured data formats for regulatory information will enable regulatory professionals to spend more time on data analysis and insights generation, particularly around the benefit-risk profile of the product (38). At the same time, the adoption of more structured benefit-risk analyses by NRAs could lead to greater harmonization of benefit-risk assessment globally and provide a framework that could be incorporated into health authorities’ benefit-risk algorithms (39).

Real-time exchange of data between companies and regulators will rely on the use of greater dynamic review processes by national regulatory authorities, as happened during the COVID-19 pandemic, and as has been developed by the FDA Oncology Center of Excellence in their Real-Time Oncology Review (RTOR) processes (40). This needs to be supported by greater harmonization and reliance between national regulatory authorities (NRA)s (see Section 6). Additionally, global harmonization and alignment of data standards will facilitate this exchange of data. Facilitating conversations between companies and health authorities will require data that is “findable, accessible, interoperable, and reusable (FAIR)” (38). Health Level Seven International’s Fast Healthcare Interoperability Resources (FHIR) system may prove to be the system of choice for sharing of regulatory information (37). It is already being utilized for sharing of health information. Integration of regulatory information with other health information utilizing a common operability system would have significant benefits. For example, real-time updates of physician prescribing software when product safety updates are approved by NRAs. It will also enable data mining and the collection of real-world data to improve the quality use of medicines and healthcare products.

Regulators are implementing procedures to ensure they capture new and emerging technologies that are not explicitly covered in their legislation such as the FDA’s emerging technology program (ETP) – established in 2014 as a way for FDA and industry to discuss potential regulatory issues regarding the development and use of a novel technology (41). New technologies, especially in manufacturing, such as continuous and modular manufacturing, use of AI models to replace empirical testing, training the model and interfacing it with advanced analytics, the development of digital twins and active process control require clear articulation of the benefit:risk balance and international standards before regulatory bodies take different perspectives on the use of such technology. National regulators should work together to ensure a smooth and harmonized integration of such technology into therapeutic product development and review.

## 4. Evolving therapeutic landscape

Rapid advances in our understanding of the molecular basis of disease are leading to the development of innovative new therapies to treat disease (42–44). This has led to a significant growth in new types of therapies in development including cell and gene therapy products, mRNA-based therapeutic products, products derived using clustered regularly interspaced short palindromic repeats (CRISPR) technology, bi-specific antibodies, nanobodies, 3D printed products (both medicines and devices), novel drug-device combination products, as well as new delivery routes such as pulmonary drug delivery (45). Future learning healthcare systems will be focused on precision medicine, along with digital health and data science (46, 47).

The Alliance for Regenerative Medicine’s recent half-year report for 2022 identified 2093 clinical trials ongoing for regenerative medicines including 968 cell therapy, 721 cell-based immune-oncology, 372 gene therapies and 32 tissue therapy products in development (1). Indeed, the pipelines of such products are moving steadily through clinical development and onto the market. RNA therapeutics have come to the fore with the rapid development of mRNA-based COVID-19 vaccines and their ability to be rapidly updated as new strains emerge (48). Nucleic acid-based therapeutics are much broader than just mRNA-based vaccines and include antisense oligonucleotides, aptamers, small interfering RNAs, microRNAs, messenger RNA and DNAzymes (49, 50).

The first clinical trial using CRISPR-edited T-cells designed to treat non-small-cell lung cancer was reported in 2020 (51). This trial did not show an objective response in treated patients but did show no severe treatment-related adverse events (51, 52). Today there are many trials in progress using gene editing techniques such as CRISPR, Zinc Finger Nucleases (ZFNs) and Transcription Activator-Like Effector Nucleases (TALENs) (53). Regulatory agencies are starting to see these products coming through development and several have been granted regenerative medicine advanced therapy (RMAT) designation by the US Food and Drug Administration (FDA) (e.g., CTX110 from CRISPR Therapeutics) or Priority medicines (PRIME) or orphan drug designation by the European Medicines Agency (EMA) (e.g., CTX001 from CRISPR Therapeutics and RP-L301 from Rocket Pharmaceuticals) (54).

Digital therapeutics is a growing area for research and development and their growth reflects the growing empowerment of patients in participating in their health and treatment decisions (55, 56). Digital therapeutics are behavioral treatments delivered online, usually via apps, that are intended to increase patients’ health outcomes. Digital therapeutics are most often categorized as software-as-a-medical-device (SAMD) and regulators are developing guidance documents on how these products will be regulated, both via international forums such as the International Medical Device Regulators Forum (IMDRF) and from individual regulatory bodies such as the Therapeutic Goods Administration (TGA), FDA, and Medicines and Healthcare products Regulatory Agency (MHRA).

These and other emerging types of therapeutics challenge current regulatory practices and regulators are responding by using horizon scanning to be more alert to the new technologies coming through development to ensure they can meet expertise gaps in the regulation of these technologies (15, 57–59). Independent horizon-scanning groups are also developing to support regulatory and policy development such as the Innovation Observatory, which is a national horizon scanning facility funded by the National Institute for Health Research in the UK. As the pace of innovation grows, we expect to see greater utilization of horizon-scanning by individual national regulatory authorities but also by consortia of regulators such as the International Coalition of Medicines Regulatory Authorities (ICMRA). The outcomes of these horizon-scanning endeavors should include focus research areas to further develop regulatory science, points-to-consider and guidance documents for developers of such technologies and inform developers of the thinking by regulators regarding identifying and managing the risks and benefits of these new therapeutic products.

The rapid development of new types of treatments, particularly high-cost curative treatments, is impacting the traditional methodologies used by Health Technology Agencies (HTA) to determine the cost:benefit analysis of new treatments (60). While not directly impacting on the benefit:risk decisions made by NRAs, a new focus on value will drive industry decisions around product development and move the decision points for value to earlier in the product development lifecycle (61). It will also encourage the inclusion of patient-reported outcomes in registration clinical trial designs to capture this information earlier for HTA analysis (62). A recent example of greater collaboration between a health authority and a health technology agency is the Innovative Licensing and Access Pathway (ILAP) in the UK. The MHRA and the National Institute for Health and Care Excellence (NICE) are working together to provide medicine developers with an innovation passport designation which provides early access to help in developing a target development profile as well as support toolkits. The regulatory professional should remain aware of changes at the interface between registration and reimbursement.

## 5. The centrality of the patient

Over the last several years we have seen a rise in the centrality of the patient in all stages of drug development to maximize alignment of product development with the needs of patients (63). The World Health Assembly has recently released a resolution on strengthening clinical trials which includes recognition of the essential contribution of trial participants and the need for inclusion of under-represented populations in clinical trials (World Health Assembly (WHA) resolution WHA75.8). The European Patients’ Academy on Therapeutic Innovation (EUPATI) is a pan-European Innovative Medicines Institute (IMI) project of 33 organizations with partners from patient organizations, universities, not-for-profit organizations, and pharmaceutical companies developed to increase the capacity of patients and patient representative groups to make meaningful contributions to medicines development and research. EUPATI has released guidance documents outlining the principles for patient involvement in research and development (64). The voice of the patient is well established in the reimbursement and pricing decisions. For example, the US Institute for Clinical and Economic Review (ICER) has a patient portal to collect patient experience information that contributes to their evidence-based reviews of healthcare interventions. Other pricing agencies, such as the Scottish Medicines Consortium and the National Institute for Health and Care Excellence (NICE) in the UK have strongly developed formal procedures for incorporation of the patient experience in their reimbursement decision-making processes.

National regulatory authorities are also looking at how best to incorporate the patient perspective into their benefit-risk assessment procedures. The MHRA’s delivery plan for 2021-2023 is focussed on “Putting the patient first” with clear actions “to embed the needs and expectations of patients” (3). The FDA has released four new guidance documents on “*Patient-Focused Drug Development Guidance Series for Enhancing the Incorporation of the Patient’s Voice in Medical Product Development and Regulatory Decision Making*” with the aim of enhancing the systematic collection of robust patient and caregiver inputs to inform product development and regulatory decision making. EUPATI has developed guidance for patient involvement in regulatory processes (65). The EMA has a well-developed process for incorporation of the patient voice into their regulatory decisions and have recently updated their framework for engagement between the EMA and patients and patient advocacy groups. One structured method for the incorporation of the patient voice into regulatory decisions, by both industry and regulators, is the use of structured benefit:risk decision making processes such as multi-criteria decision analysis (39, 66, 67).

## 6. Global regulatory harmonization and convergence

Mirroring the advances in complex therapeutics, regulatory authorities are increasingly seeking to work together through various mechanisms such as harmonization, convergence, reliance, collaborative review and work-sharing and this has been accelerated by the global COVID-19 pandemic (68–70). They are working through collaborative fora such as the International Pharmaceutical Regulators Programme (IPRP), ICMRA and IMDRF to identify areas for potential synergies and address regulatory and safety challenges strategically. Underpinning these endeavors is greater transparency, being one of the main principles on which good regulatory practices is based and GRP is critical for the cooperation of regulatory authorities (71, 72). Harmonization is defined as the process of integrating national and international standards to facilitate efficiencies in global drug development and regulation (73). A well-known example is the integration of the ICH guidelines by national regulatory authorities (74). In the devices area, harmonization was initiated through the Global Harmonisation Task Force (GHTF) which has now been superseded by the IMDRF. Convergence may be defined as the process whereby the regulatory requirements across different countries become more aligned due to the adoption of global standards, documents and best practice (73, 75). An example of regulatory convergence is the establishment of the Regulatory Harmonization Steering Committee of the Asia-Pacific Economic Cooperation (APEC), which was established in 2008 to drive convergence of regulatory requirements and harmonization of registration management across the APEC member states (76–78). Another example is the Pan American Network for Drug Regulatory Harmonization (PANDRH), which is an initiative of the national regulatory authorities within the pan-American region, and the Pan-American Health Organisation (PAHO), that supports the processes of pharmaceutical regulatory harmonization in the Americas, within the framework of national and sub-regional health policies and recognizing pre-existing asymmetries (79). The WHO defines reliance as “*the act whereby the regulatory authority in one jurisdiction may take into account or give significant weight to work performed by another regulator, or trusted institution, in reaching its own decision.*” (71).

Reliance may take many forms and reflect varying degrees of application in recognizing or taking account of the assessments, decisions or any other authoritative information available from other authorities and institutions. For example, the Australian TGA has implemented the Comparable Overseas Regulator pathways to such effect. The acceptance of the Certificate of Pharmaceutical Product (CPP) by some national regulatory authorities is also an example of reliance. Some regulatory authorities are using reliance pathways to enable an abridged evaluation process, hence speeding up the review process in their countries. The EMA introduced a pilot project called “OPEN” during the COVID-19 pandemic to allow international participation in their scientific evaluation process by other regulatory agencies with which they had confidentiality arrangements, another example of reliance and cooperation between international regulatory agencies. Participants included Health Canada (Canada), Japan Ministry of Health, Labor and Welfare/Pharmaceuticals and Medical Devices Agency (MHLW/PMDA), Swissmedic, Therapeutic Goods Administration (TGA) and the World Health Organization (WHO). Representatives from the participating agencies could attend the Committee for Human Medicinal Products (CHMP) and emergency taskforce meetings for COVID-19 related treatments and vaccines. It remains to be seen whether this initiative will be extended to other therapeutic areas post-pandemic.

A collaborative review program developed by the US FDA, Project Orbis, has the goal of accelerating regulatory approval of innovative oncology medicines among participating countries (7). Initial evidence indicates that the program is achieving its intended purpose and the number of participating countries has increased (7, 80).

Continuing along the evolution of harmonization, convergence and reliance, we have seen the implementation of a work-sharing arrangement between a number of comparable, mid-sized national regulatory authorities with the establishment of the ACCESS Consortium between the regulators in Australia, Canada, Singapore, Switzerland and the United Kingdom. These countries are also participants in Project ORBIS. Work sharing has developed over time, following extensive information sharing and confidence building between the participating regulators to reach the point that participating regulatory authorities divide the Modules for review under confidentiality agreements and memoranda of understanding between the participating authorities. Each participant retains their own sovereign decision-making ability, but the process lightens the workload of the regulators and enables sharing of expertise across different geographies (8). These initiatives all point to greater collaboration and cooperation between national regulatory authorities to enable them to tackle the challenges of regulating new, innovative therapies and ensuring accelerated access to patients in their countries.

## 7. Challenges arising from these identified trends

There are a number of challenges associated with each of these trends that will need to be dealt with as they impact drug development, medical practice and the role of the regulatory professional. The major challenges with the rise of AI and ML are around governance and ethics. Ethical concerns include the protection of human autonomy, well-being and privacy, ensuring transparency and explainability of the deep learning models used in AI and ML, ensuring responsible and accountable use of these technologies, ensuring inclusiveness and equity to reduce bias and promoting responsive and sustainable AI (81). Governance concerns include the governance of data, including how informed consent is obtained for data used to train AI/ML, how data are de-identified and individual privacy is protected, how data are shared, managed and controlled, cybersecurity and assignment of intellectual property rights (81). Strong governance and ethical frameworks will be needed to increase confidence in the use of AI/ML and quality assurance and auditing processes will need to be well established within organizations relying on these systems. Regulatory professionals will need to be confident in explaining these technologies, ensuring they are compliant with government legislation and liaising with regulators in the registration of AI/ML-based therapeutic products. They will need to be able to integrate such systems into their formal benefit-risk decision-making processes and communicate that clearly within their organization and with external stakeholders.

Digital disruption challenges organizations with respect to upskilling and reskilling their staff (82). The main drivers of successful digital change management within organizations will be cultural challenges – implementing a culture of continuous learning, encouraging an agile mindset in employees and bringing all staff along on the digital transformation journey. This requires organizations to review their leadership styles, staff competency profiles that will be needed to successfully transition the organization, and incentives to encourage continual professional development of their staff. Entrenched mindsets and infrastructure will need to be examined and changed to support staff during this transition. Another challenge is the increasing pace of change and staff feeling burnt-out and incapable of further adaptation. The risk and impact from the loss of highly technically experienced staff who may struggle with new role expectations, will require the implementation of a strong supportive environment. Creating a sustainable workforce that is effective in a hybrid working environment will require strong organizational change management to develop an organization that ensures staff have the desire to change and develop to meet the future business needs (83). A mentorship program where technically adept staff members are supporting less confident staff in their transition will help overcome some of these issues. Supporting staff to undertake both internal and external learning opportunities to build skills and experience with new systems and ways of working will be essential.

Staying up to date with the changing therapeutic landscape and the development of novel therapeutics is a challenge for us all. One way that individuals can try to remain current is coming together to share learnings about new technologies and therapies within their specialization. Attending relevant scientific conferences, taking university courses or micro-credentials on certain topics will supplement informal on-the-job learning. Integration of the patient perspective into all stages of drug development requires a conscious decision by management within organizations involved in therapeutic product discovery and development. It will also require the regulatory professional to stay at the forefront of the thinking by regulatory agencies and legislators.

While we see global harmonization, convergence and reliance as increasing, it will do so in a background of increased nationalism because of the COVID-19 pandemic, the war in Ukraine resulting in global energy shocks, and climate destabilization. Advanced economies will need to ensure that they bring less developed economies along the path toward greater harmonization, convergence and reliance to facilitate greater equity of access to the benefits of new healthcare technologies and therapies. Advanced economies will also need to support infrastructure changes within the regulatory agencies of developing economies to facilitate information exchange. Finally, focused training and exchange of regulatory personnel between agencies will help to foster regulatory best practice globally.

## 8. Skills for the future regulatory affairs workforce

These developments in healthcare, medicine and the pharmaceutical and medical device industry will impact the regulatory affairs team. The traditional heavy ‘task’ based workload will evolve with digital solutions and automation to require broader strategic leadership skills. It is therefore vital that regulatory professionals are equipped with the skills, knowledge, and mindset to develop themselves in order to advance their professional lives. The current world of work is said to be volatile, uncertain, complex, and ambiguous (VUCA) and the global pandemic has resulted in a “new normal” world of work where these factors are amplified (84–86). The World Economic Forum has identified a number of important skills for the future of work including analytical thinking and innovation, active learning, complex problem-solving, critical thinking and analysis, creativity, originality and initiative (87). Additional skills identified for the future of work include leadership and social influence, the ability to utilize, design and monitor technology, resilience, an ability to tolerate stress, flexibility and reasoning, problem-solving and ideation (87). The Institute for the Future identified additional skills in sense-making, cross-cultural competency, virtual collaboration and trans-disciplinarity as essential for the future of work (88). These skills will all be important in the pursuit of careers in the pharmaceutical and medical technology industry.

**FIGURE 2:**
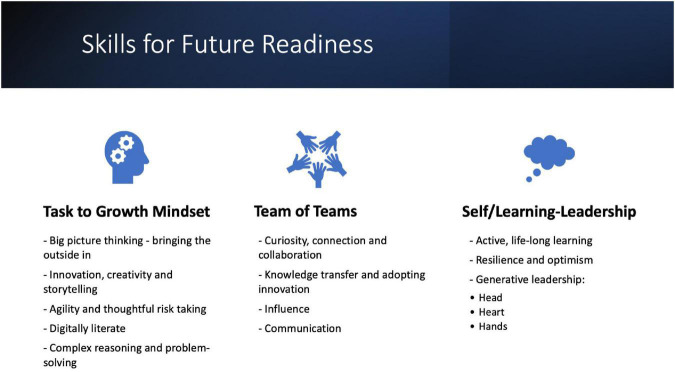
Skills required for future readiness for regulatory affairs professionals.

Given the major impact of digital transformation, regulatory professionals should focus on their digital literacy skills such as being confident in using dashboards and cloud-based platforms for data visualization, understanding how data are acquired, processed, analyzed and used for predictive purposes, and developing statistical data analysis and data mining skills (29). Beyond this there is a need to increase competencies in complex reasoning and problem-solving, adaptive thinking, agility, communication and teamwork and leadership and initiative – all elements of what is known as 21st century skills (89, 90) (Figure 2). Generative leaders who strive to leave the world a better place than they found it lead equally with their head, their heart and their hands to unlock the greatest value. This requires a bold vision for the future by reimagining and reinventing ways of working to serve all stakeholders (leading with the head), building a culture that inspires and enables people to do their best work (leading with the heart) and executing through and empowering teams (leading with the hands) (91). These elements together can lead to transformational change, a necessary requirement for thriving in the VUCA world of work. Professionals who take control of their own learning will be able to keep pace with the transformations happening in the industry in general and regulatory affairs specifically and position themselves for future growth opportunities.

Regulatory professionals should develop a plan for their continual professional development and learning in conjunction with their managers, after reviewing their individual competencies and identifying gaps. They may seek a mixture of formal education, short courses or micro-credentials or informal learning opportunities to upskill (92). Short term assignments working in different areas also helps to deepen knowledge and connection and broaden perspectives. This aligns with a growth rather than a task mindset to enable a solution focused way of working. Besides further education and training to develop the necessary skills, regulatory professionals can ensure that they are regularly assessing trends that may impact their work, for example, by regularly reviewing activities and outputs from international regulatory bodies such as ICMRA, IMDRF, IPRP, ICH, and WHO, and keeping up to date on the latest communications from industry bodies in major jurisdictions such as the International Federation of Pharmaceutical Manufacturers and Associations (IFPMA), European Federation of Pharmaceutical Industries and Associations (EFPIA), Pharmaceutical Research and Manufacturers of America (PhRMA), Association of the British Pharmaceutical Industry (ABPI) and other local industry associations. Of course, keeping up to date with what each of the regulators are doing is also vitally important. Regulatory professionals should review updates to strategic plans from regulators to identify future directions for regulatory agencies, as well as updates to legislation. The impacts of major policy changes should be considered, such as the Pharmaceutical Strategy for Europe (93) or updates to country-specific national medicines policies, such as the current update to the National Medicines Policy in Australia (94, 95). The implementation of such policies may have implications beyond just the country or region involved in the initiative. Reviewing such changes will help regulatory professionals identify international discussions and where various drivers for change are coming from. Finally, regulatory professionals should keep up to date with relevant government reviews that may impact their organizations and industry.

## 9. Conclusion

The future of the regulatory affairs profession is exciting and will be shaped by several factors, particularly digital disruption. Digital disruption is pervasive and impacting all aspects of work, accelerated by the COVID-19 pandemic and the rapid growth in complexity and capabilities of machine learning and artificial intelligence algorithms. Other trends impacting the future of this profession include the rapid advances in the scientific understanding of disease, leading to new types of therapies to treat or even cure some diseases. A renewed focus on the centrality of the patient and involvement of the patient in all aspects of therapeutic product development, will ensure that products add value to patients’ lives. The global regulatory environment has changed dramatically over the past several years with a greater emphasis on strategic collaborations, harmonization, and convergence between national regulatory authorities and this trend is likely to continue. As these factors begin influencing the work of the regulatory professional, drug development and medical practice, it would be interesting to review their impact in a few years’ time. These changes require upskilling of regulatory affairs professionals and a change from a task-focused mindset to a growth mindset, where individuals take control of their professional development, are agile and adopt a perspective of continual learning to ensure they can maximize their influence on product development for the betterment of their society.

## Author contributions

OC and HC conceived the idea for the manuscript. OC drafted the manuscript. HC provided the feedback on drafts. Both authors approved the final manuscript.
